# Too Hot to Sleep? Sleep Behaviour and Surface Body Temperature of Wahlberg’s Epauletted Fruit Bat

**DOI:** 10.1371/journal.pone.0119419

**Published:** 2015-03-16

**Authors:** Colleen T. Downs, Adwoa Awuah, Maryna Jordaan, Londiwe Magagula, Truth Mkhize, Christine Paine, Esmaella Raymond-Bourret, Lorinda A. Hart

**Affiliations:** School of Life Sciences, University of KwaZulu-Natal, P/Bag X01, Scottsville, Pietermaritzburg, 3209, South Africa; University of Western Ontario, CANADA

## Abstract

The significance of sleep and factors that affect it have been well documented, however, in light of global climate change the effect of temperature on sleep patterns has only recently gained attention. Unlike many mammals, bats (order: Chiroptera) are nocturnal and little is known about their sleep and the effects of ambient temperature (T_a_) on their sleep. Consequently we investigated seasonal temperature effects on sleep behaviour and surface body temperature of free-ranging Wahlberg’s epauletted fruit bat, *Epomophorus wahlbergi*, at a tree roost. Sleep behaviours of *E*. *wahlbergi* were recorded, including: sleep duration and sleep incidences (i.e. one eye open and both eyes closed). Sleep differed significantly across all the individuals in terms of sleep duration and sleep incidences. Individuals generally spent more time awake than sleeping. The percentage of each day bats spent asleep was significantly higher during winter (27.6%), compared with summer (15.6%). In summer, 20.7% of the sleeping bats used one eye open sleep, and this is possibly the first evidence of one-eye-sleep in non-marine mammals. Sleep duration decreased with extreme heat as bats spent significantly more time trying to cool by licking their fur, spreading their wings and panting. Skin temperatures of *E. wahlbergi* were significantly higher when T_a_ was ≥35°C and no bats slept at these high temperatures. Consequently extremely hot days negatively impact roosting fruit bats, as they were forced to be awake to cool themselves. This has implications for these bats given predicted climate change scenarios.

## Introduction

Sleep is a state that can be defined in terms of behavioural responses of sustained immobility with greatly reduced responsiveness to external stimulation, and in terms of physiological responses such as a reduction in metabolism, body temperature etc. [1,2]. It plays an important role in mammals, which can spend a quarter or more of their lives sleeping [3,4]. Sleep has various profitable effects including memory restoration and retention, functionality of the immune system, and conservation of energy [5,6,7,8,9]. Consequently, sleep deprivation is known to negatively impact the cognitive responses of the brain [10,11], various aspects of physiology [12] and vigilance [10].

The amount of sleep needed and the ability to fall asleep in mammals differs between individuals and species [11,13]. Many other factors also influence sleep patterns, duration and intensity e.g. light cycle, food availability, noise conditions, predation risk and temperature [2,6,14]. Sleep patterns and duration are highly susceptible to ambient heat [15] and duration generally decreases particularly with heat stress [16,17,18].

Over the past century, global average temperature has increased by almost one degree and is still increasing at a rapid rate [19]. The 1990’s were the warmest decade, and 1998 the warmest year on record [20]. In eastern South Africa mean annual maximum and minimum temperature increased by 1 and 3°C respectively between 1951 and 1990 [20]. It is predicted that the current trends of climate change will lead to further increases of 2–3°C within the next fifty years [21,22]. In addition to increased ambient temperatures (T_a_), climate change has resulted in changed rainfall patterns, with some areas of the world experiencing extreme weather conditions, including extended periods of drought or extreme heat [23]. Accelerated climate change is likely to affect abundance, natural distribution and behaviour of many species [19,24,25]. Consequently climate change may affect sleep behaviour, and have consequences on behaviour, physiology, distribution, and ultimately fitness.

There are considerable data on sleep patterns under heat stress in humans and rats [15,18,26,27,28]. However, to our knowledge, the effect of temperature on sleep behaviour of Chiroptera has not been studied. Fruit bats have no sweat glands so it is difficult to lose heat [29] and thus they are more vulnerable to high temperatures where they cannot dissipate sufficient heat to prevent increased and lethal body temperatures [30]. As fruit bats usually roost outside, they cannot benefit from buffered microclimates in insulated roosts and extreme high T_a_s have been recorded to cause mortality in some species [19,30,31].

Wahlberg’s epauletted fruit bat (*Epomophorus wahlbergi*) occurs widely in Africa, including southern Africa’s east coast [32,33]. These bats are found in savannah, woodland and forest margins where fleshy fruits are available and also occur in treed peri-urban areas [31,32]. They forage for fleshy fruits and nectar during the night. Individuals generally roost in trees or under the eaves of buildings [32,33]. Roost sites are therefore exposed to unbuffered ambient temperatures that vary throughout the day [31].

We investigated the effects of seasonal variation in T_a_ on sleep patterns and surface body temperatures (T_skin_) of free-ranging *E*. *wahlbergi*. We expected that this nocturnal species would spend most of the daytime inactive period sleeping. In summer and winter they become heterothermic (body temperature that varies) during the day where body temperatures fluctuate in response to environmental temperature, but do not employ true torpor [30] which is the dormant, inactive state of a hibernating or aestivating animal. At high temperatures they generally exhibit thermoregulatory behaviours to dissipate heat including licking their fur, spreading their wings and panting [30]. Consequently we expected higher T_a_, particularly >35°C, would affect the bats’ sleep duration negatively. We predicted that the bats would sleep more in winter than in summer, and that their T_skin_ would be lower in winter than summer. We expected bats to increase thermoregulatory behaviours that dissipate heat at higher T_a_s, especially in summer.

## Methods

**Fig 1:**
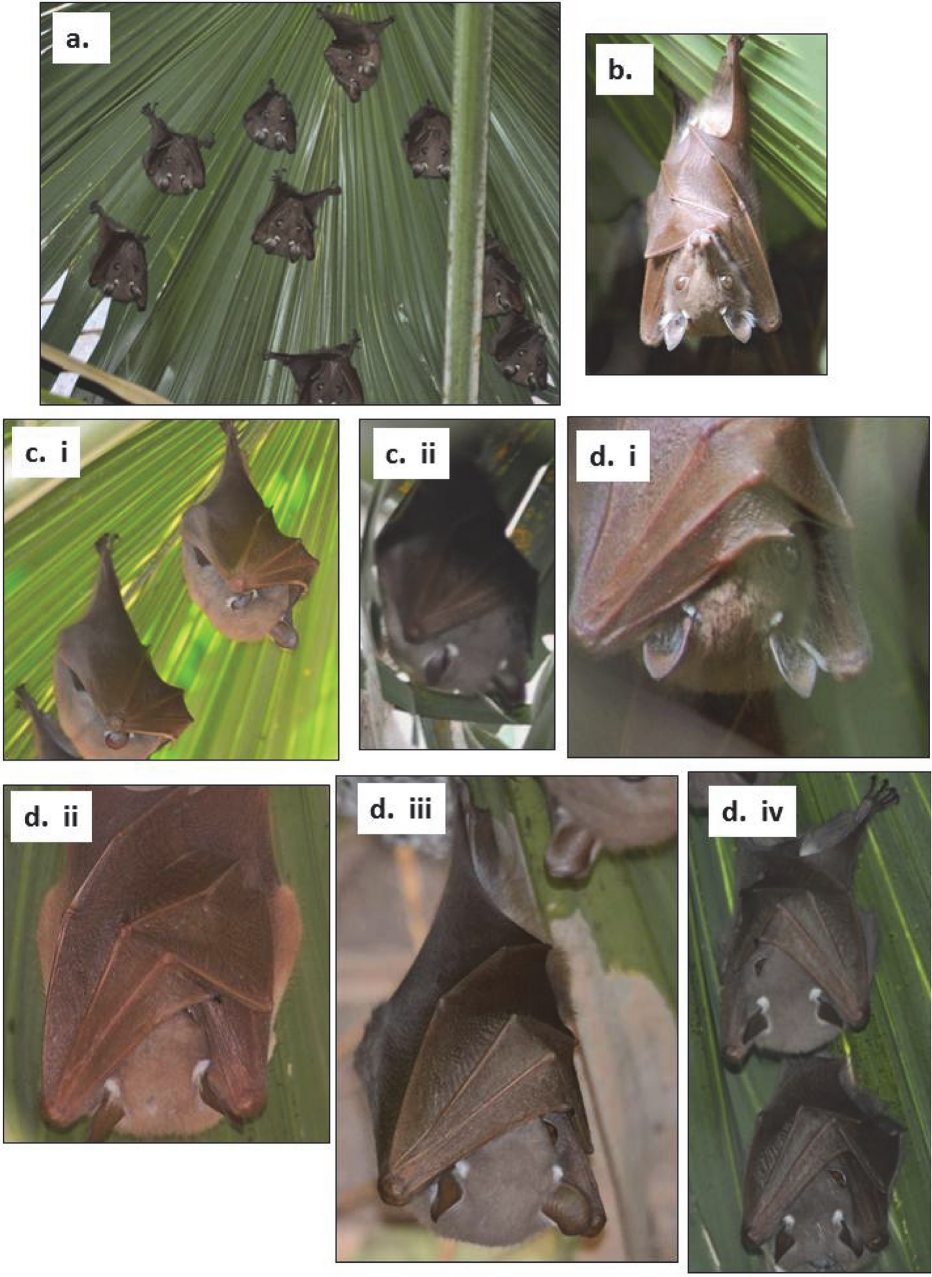
Photographic examples of *E*. *wahlbergi* roosting and showing different non- and sleep behaviours (see methods) where a. bat roost; b. awake bat with both eyes open; c. bats sleeping with both eyes closed, (bats sleep usually with wings wrapped around and covering both eyes), and d. bats sleeping with one eye closed. (Captured using a Nikon D5000 digital camera, copyright CT Downs).

As little is documented on sleep of fruit bats, we first examined how *E*. *wahlbergi* sleep under captive conditions in terms of sleep posture (position of the body and body parts), type and duration. We had previously captured six *E*. *wahlbergi* in mist nests near the University of KwaZulu-Natal (UKZN, 29° 62’ 154”S, 30° 39’ 641”E), Pietermaritzburg campus (under permit from Ezemvelo KZN-Wildlife and animal ethics permission from UKZN 024/13/Animal) and kept them in outside aviaries (see [34] for details). Bats were moved to a controlled environment in the Animal House at UKZN, Pietermaritzburg campus (25°C, 12L:12D) during August. Bats were kept individually in cages (75 x 51 x 80 cm). Each evening before dark, they were provided with 20% sugar water and fresh fruit. We used a VIVOTEK IP7330 Network Bullet Camera fastened to the side of the cage and linked to a computer to record behaviour continuously during the day when bats were inactive. Each bat was recorded for two days before being released to the outside aviary. From the recordings we measured the type of sleep posture, length of time each bat slept, or was awake each day. When resting, bats had either both eyes open, or both eyes covered by their wings or one eye covered by a wing. When bats had their wings over both eyes we assumed they were sleeping with both eyes closed. We assumed that bats with one eye closed or hidden behind the wing were sleeping with one eye open (Fig. 1, see discussion). The particular sleep type exhibited by each individual was recorded for each sleep episode during each day. We used a nonparametric Wilcoxon matched pairs test to compare the percentage of time the fruit bats slept or were awake, as well as the percentage time they spent sleeping with both eyes closed compared with one eye open.

We also observed free-ranging *E*. *wahlbergi* during the day at a natural roost in a single palm tree (*Borassus* sp.) between two buildings on the UKZN, Pietermaritzburg campus (29° 62’ 154”S, 30° 39’ 641”E) during summer (January 2013, n = 15 days) and winter (June and July 2013, n = 20 days). The roost tree is located between two multi-story buildings (6 m apart) with walkways between the different floors and buildings. The bats were therefore habituated to humans walking past and stopping to look at them. We took measurements during university holidays, when the traffic along the walkways was minimal to reduce any potential impact on bats’ behaviour.

During both seasons we observed free-ranging *E*. *wahlbergi* hourly, initially from a distance (± 5 m) and then close up (± 2 m) using binoculars (Asahi PENTEX, Prism binoculars, 8 x 30 mag., Japan) to measure sleep parameters (posture, type and duration). Every hour, between 08h00 and 16h00, the number of bats roosting, their sleep type (i.e. awake or sleeping with one or both eyes closed) and evidence of thermoregulatory behaviour (i.e. extension of wings, panting and fur licking) by any of the bats (presence/absence) were noted. We assumed that bats with one eye closed or hidden behind the wing were sleeping with one eye open (Fig. 1). As the number of bats observed sleeping from a distance did not differ significantly with observation distance, we only present data for close up observations. Additional comments on disturbances at the roost were also noted such as strong wind or the presence of other animals in / near the roost.

**Fig 2:**
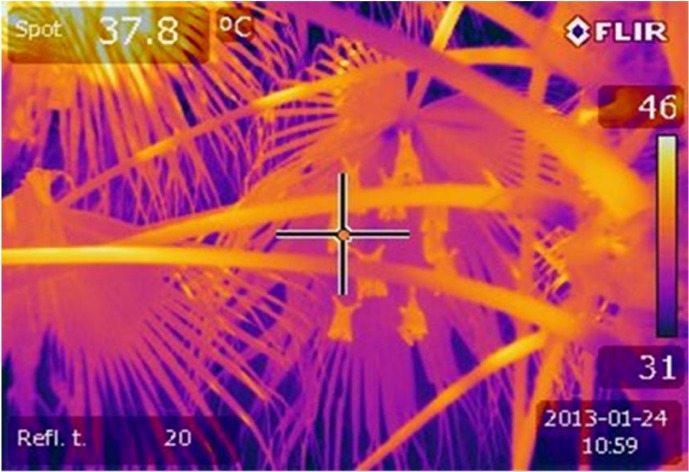
Thermal image of *E*. *wahlbergi* roosting in the fronds of a *Borassus* sp.

We took hourly images of *E*. *wahlbergi* using a calibrated thermal camera (Fig. 2, FLIR E60, FLIR Systems Inc., Estonia; temperature range −20 to 650°C, thermal sensitivity <0.05°C at 30°C) to measure T_skin_. The point taken for T_skin_ was on the ventral abdominal surface of an individual. We recorded maximum T_a_ each hour using a hand held weather tracker (Kestrel 4000, Nielsen-Kellerman, USA) mounted in the shade within 2 m of the roost.

As the number of *E*. *wahlbergi* at the roost varied from day to day, we used the percentage of bats sleeping rather than the actual number of bats sleeping. We assumed that the state of a bat (i.e. sleeping / awake / licking / open mouth / spreading of wings) was independent for each hourly observation.

Descriptive statistics, General Linear Models Repeated Measures Analysis of Variance (RMANOVA) and Kruskal-Wallis tests were conducted using Statistica (Statsoft, version 7, Tulsa, USA). All analyses were made using 95% confidence intervals and all data are presented as mean ± SE.

## Results

### Laboratory

In the controlled conditions at 25°C during rest phase, *E*. *wahlbergi* (n = 6) showed a typical roosting posture of hanging by their feet from the cage roof, and with their wings folded. They spent significantly more time awake then asleep (71.1% of day time awake vs. 28.8% sleeping; P > 0.05). Most sleeping (79.2%) occurred with both wings over the eyes while some (10.8%) sleeping occurred with one eye open (one eye closed or hidden behind the wing). No thermoregulatory behaviours to dissipate heat were observed.

### Ambient temperature and roosting bats

**Fig 3:**
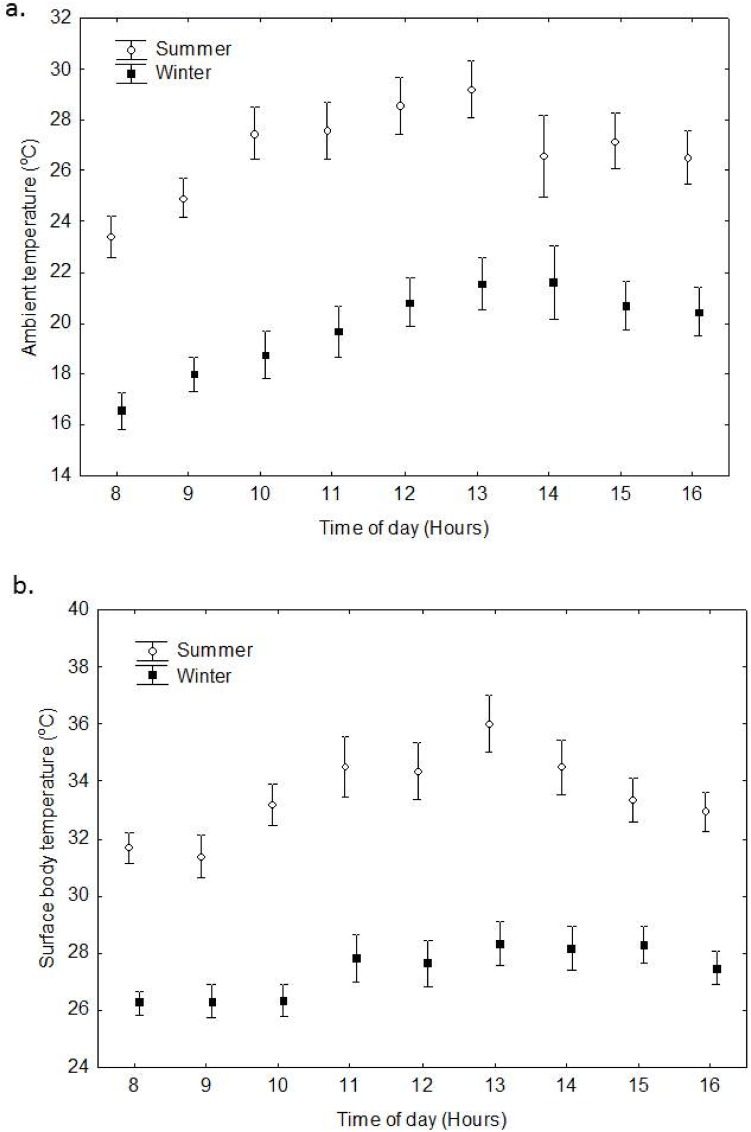
Mean hourly variations in a. ambient temperature, and b. surface body temperature of free-ranging *E*. *wahlbergi* at the roost during summer (January) and winter (June—July). (Vertical bars denote ± SE).

There were no significant seasonal differences in T_a_ at the outside roost when combined with time of day (RMANOVA F_(8, 256)_ = 1.689, P = 0.101, Fig. 3A). However, when only season and T_a_ were compared, season did have a significant effect on T_a_ (RMANOVA, F_(1, 32)_ = 32.292, P > 0.05). Similarly T_a_ varied significantly with time (RMANOVA, F_(8, 256)_ = 14.678, P > 0.05, Fig. 3A). During summer, min—max T_a_ ranged from 18.6–41.0°C, while during winter T_a_ ranged between 12.6–29.6°C.

There was no significant difference in number of *E*. *wahlbergi* at the roost between seasons and with time of day (RMANOVA, F_(8, 256)_ = 1.08, P = 0.38). Mean number of roosting bats per hour per day in winter ranged from 15.5 ± 1.35 to 16.1 ± 1.37, and number of bats ranged from 8–24 (n = 20 days). In summer mean number of roosting bats per hour per day ranged from 18.9 ± 1.52 to 20.3 ± 1.52 and number of bats at the roost ranged from 11–27 (n = 15 days). On most days the number of bats at the roost remained the same and in the same position throughout the day. On a few occasions 2–3 bats left the roost during the day, mostly due to a disturbance.

### Surface body temperature

**Fig 4:**
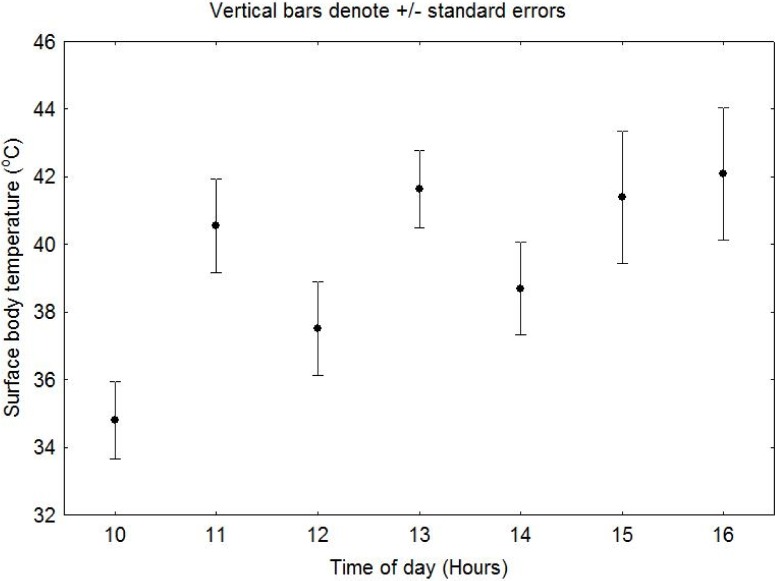
Mean hourly variations in surface body temperature of *E*. *wahlbergi* when roost ambient temperatures ≥ 35°C in summer. (Vertical bars denote ± SE).

There were no significant seasonal differences in T_skin_ when combined with time (RMANOVA F_(8, 232)_ = 1.267, P = 0.0.262, Fig. 3B). However, when only T_skin_ and season were compared, T_skin_’s were significantly lower in winter compared with summer (RMANOVA, F_(1, 29)_ = 65.843, P > 0.05, Fig. 3B). T_skin_ also varied significantly with time through the day (RMANOVA, F_(8, 232)_ = 7.46, P > 0.05, Fig. 3B). In summer when T_a_s were > 34°C, the mean T_skin_s of bats were high (>37°C) and all bats tried to dissipate heat behaviourally (Fig. 4). The maximum T_skin_ recorded in summer was 46.1°C.

### Sleeping behaviour in the wild

**Fig 5:**
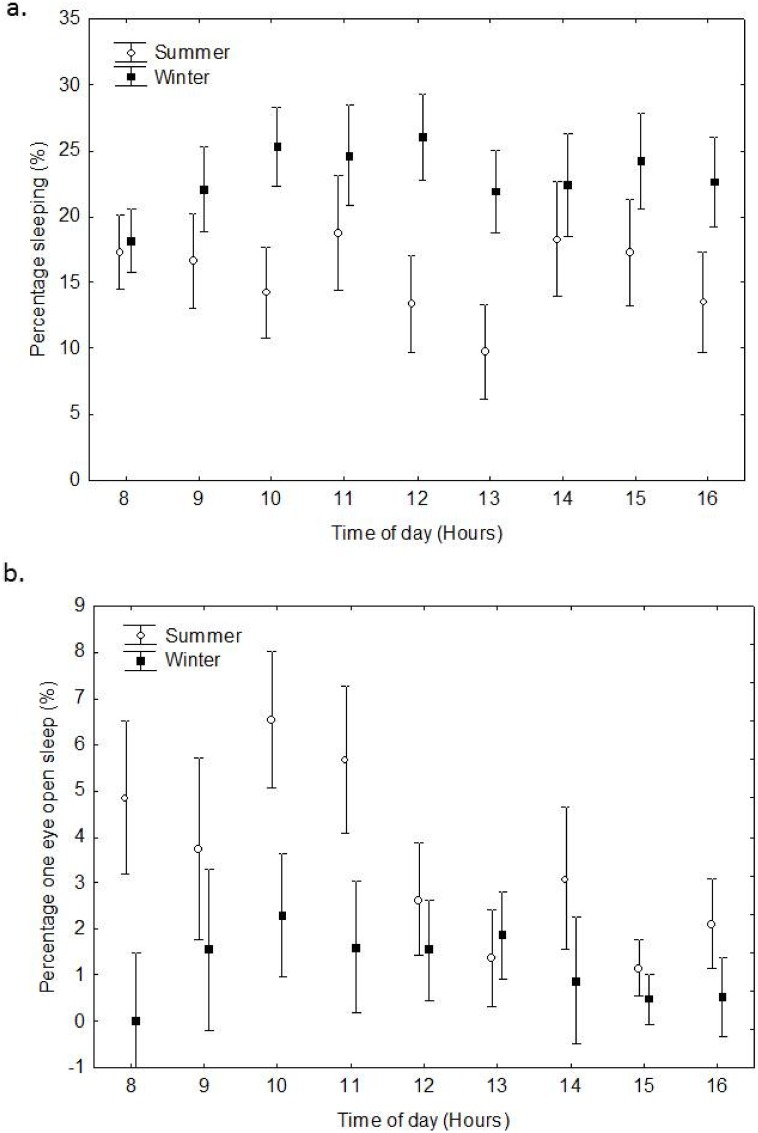
Mean percentage of sleeping *E*. *wahlbergi* a. throughout the day, and b. using one eye closed sleep, in summer and winter at the roost. (Vertical bars denote ± SE).

As in captivity, all *E*. *wahlbergi* showed a typical roosting posture of hanging by their feet from a leaf frond, with their wings folded. The proportion of *E*. *wahlbergi* sleeping at the roost was generally low in both seasons and at any given hourly sample (Fig. 5A). There was no significant difference in number of sleeping bats in summer compared with winter and time during the day (RMANOVA, F_(8, 256)_ = 1.13, P = 0.342, Fig. 5A,). The percentage of bats sleeping in summer ranged from 9.7 ± 3.59% to 18.8 ± 4.33% compared with winter 18.2 ± 2.44% to 26.1 ± 3.24% (Fig. 5A). In winter bats generally slept for longer periods of time, and more often than in summer. In summer, fewer bats slept during midday (Fig. 5A) as individuals spent much of the day performing behaviours to cool themselves. No bats were observed sleeping when T_a_ exceeded 35°C. In contrast, during winter, the percentage of *E*. *wahlbergi* sleeping increased from 09h00 to 12h00 as T_a_ increased (Figs. 2A, 5A).

In both seasons, more *E*. *wahlbergi* were observed sleeping with both eyes closed than with one eye open. The latter was low in both seasons (Fig. 5B) and did not differ significantly with season or time (RMANOVA, F_(8, 256)_ = 1.29, P = 0.247). In summer, percentage of sleep with one eye open ranged from 1.2 ± 0.60% to 6.5 + 1.48% while in winter it ranged from 0.0 ± 1.48% to 2.3 ± 1.32% (Fig. 5B). Use of one eye open sleep varied throughout the day. The first hours after nightly activity, sleep with both eyes closed was more often observed. Thereafter, there was a peak in one eye open sleep around 10h00, which diminished gradually as the day progressed.

### Behavioural thermoregulation

The bats did not huddle in either season. In summer, as T_skin_ exceeded 34°C, most *E*. *wahlbergi* in the roost began to lick their wings and heads. In summer a few bats in the roost began licking themselves at T_skin_ between 31 and 33°C. Some bats changed positions and moved from branches that were in direct sunlight to branches that offered more shade in addition to the shade provided by the buildings. At T_skin_ exceeding 40°C, most (>80%) bats continued to lick themselves occasionally and some opened their mouths, possibly panting. The incidence of bats showing licking behaviour, panting or spreading their wings was greater when T_a_ was higher. Generally these thermoregulatory behaviours were observed was above 31°C. No cooling behaviours were observed in winter, instead all bats kept their wings wrapped around their bodies.

## Discussion

We found that both captive and free-ranging *E*. *wahlbergi* slept relatively little during their inactive phase. The free-ranging *E*. *wahlbergi* slept less in summer compared with winter. On hot days in summer the bats cooling strategies affected sleep behaviour.

The amount of sleeping by roosting *E*. *wahlbergi* differed from two Asian fruit bat species, the greater short-nosed fruit bat (*Cynopterus sphinx*) and the lesser dawn fruit bat (*Eonycteris spelaea*), which spend on average 62.5% of the day sleeping [35]. Small animals typically have shorter sleep cycles than larger animals [4] and animals with high metabolic rates for their body size generally have short sleep durations [36]. *Epomophorus wahlbergi* weigh 80–120 g [31] and their mass-specific basal metabolic rate is 137% (winter) and 106% (summer) predicted from allometric scaling for Chiroptera [30]. The T_skin_ we measured supports the contention that these bats do not use torpor and are heterothemic [30]. Consequently, torpor does not explain the differences in amount of sleep in winter compared with summer.

Although *E*. *wahlbergi* slept relatively little, displaying short intermittent sleep bouts, they were generally immobile in their typical hanging posture throughout the day. They exhibited both two eyes closed and one eye closed sleep types. No sleeping with one eye closed has been reported in other fruit bat species [35]. The discovery that these bats spend some time sleeping with only one eye closed is potentially interesting given that this phenomenon has been described only for birds and marine mammals [2,37,38,39,40,41,42]. In these groups, unilateral eye closure is associated with unihemispheric sleep based on data from electroencephalogram and occurs when one hemisphere of the brain shows waking electroencephalographic activity, while the other shows slow-wave sleep activity [41]. However, until the definitive link between behaviour and brain state has been confirmed, our observations only provide indirect evidence for the possibility that these bats might be the first non-marine mammal to exhibit unihemispheric sleep. Unihemispheric-short-wave-sleep allows recovery from sleep deprivation, to breathe underwater and to avoid predation while sleeping [39,40,41]. In birds it may have evolved to enable anti- predator vigilance while sleeping [39,41]. Bats in exposed roosts may be exposed to similar predation risks and thus it is plausible that they have evolved an analogous strategy. Vervet monkeys *Chlorocebus pygerythrus* and African crowned eagle (*Stephanoaetus coronatus*) have been observed hunting roosting *E*. *wahlbergi* during the day (CTD unpublished data). In Costa Rica a number of different monkey species also hunt bats during the day (C. Schoeman pers. obs.). *Epomophorus wahlbergi* in urban environments roost in trees or under the eaves of buildings and change daytime roosts regularly [31]. As these bats are hunted by predators during the day, unihemispheric sleep in bats may be likely and may have evolved to reduce predation risk just as it has in marine mammals and birds.

The use of one eye closure sleep by *E*. *wahlbergi* supports the anti-predation hypothesis for this type of sleep during the day. However, given that this form of sleep was used more in summer and relatively little, it suggests there could be alternative functions. When *E*. *wahlbergi* slept, they used less one eye closure than two eyes closed, which is comparable to that observed in birds (17%), based on eye closure only [39].

Most bats are nocturnal with little diurnal activity. Various reasons for this have been proposed including: competition for food and habitat with diurnal birds, predation, and the risk of hyperthermia [43]. It has been shown in several species that an increase in the size of the group results in decreased individual vigilance [44,45] and an increase in sleep duration [46]. We found that summer T_a_, particularly >34°C, had the greatest impact on the percentage of *E*. *wahlbergi* sleeping. High temperatures promote sleep and drowsiness in several species of mammals [15,27,47], while it has been associated with an augmentation of wakefulness in other studies [17,18]. We demonstrate that heat negatively affected *E*. *wahlbergi* sleep. This is most likely due to the fact that bats, despite a large capacity of acclimatization, have poor thermoregulatory strategies to dissipate heat at high temperatures [30,48,49]. It has been suggested that flying fruit bats risk overheating in extreme summer conditions promoting diurnal inactivity [42,50]. Furthermore *E*. *wahlbergi* have no sweat glands and so it is difficult for them to offload heat via evaporative water loss compared with most other mammals [29,49]. Consequently in summer when Ta’s reached 35°C or more because of the bats’ limited ability to dissipate heat they employed cooling behaviours and slept little. Also given that they roost in foliage they are more exposed to solar radiation and are therefore more likely to experience heat stress [50,51]. Mortality has been reported in this species on extremely hot days and exposure to T_a_’s greater than 40°C [30]. Similarly cases of mortality of fruit bats have been observed in India, Australia and South Africa on extremely hot days [19,30,51,52,53,54]. For example, in 2014 more than 2000 flying foxes died from a heat wave in Ipswich, Queensland Australia [54].

In summer, T_skin_ of *E*. *wahlbergi* increased with an increase in T_a_ and they were hyperthermic when T_a_ reached 35°C or more. Presumably to lower their T_skin_, *E*. *wahlbergi* used cooling behaviours on hot days, including licking their heads, spreading their wings and panting. Licking the fur generates cutaneous evaporative heat loss and plays an important role in thermoregulation in many mammal species, including bats [49,55,56]. Panting in the greater spear-nosed bat (*Phyllostomus hastatus*) of South America, leads to the loss of 14% of heat load through respiratory evaporation [57]. Bats also rely largely on their wings for heat exchange [57,58]. Their thin wing surface membrane possesses numerous blood vessels which can be used to offload heat when the wings are stretched [29,57,58]. Some Australian flying fox species lick their wings to increase evaporative cooling [19]. Bats can dehydrate rapidly at high T_a_’s due to excessive salivation, rostrum’s glands secretions, eye fluid extrusion and evaporative water loss from skin which is not perfectly permeable to water, especially the patagial membrane [30,59,60]. Furthermore *E*. *wahlbergi* reproduce during the summer and mothers generally roost hanging with their young attached [32,33] and so can be further affected by extremely hot days so sleeping less if having to cool off. It would also be more difficult to dissipate heat using thermoregulatory behaviours i.e. extension of wings and fur licking.

With global warming and the increasing frequency of hot days, it is expected that bats like *E*. *wahlbergi* will spend less time sleeping on these extremely hot days and more time employing cooling behaviours to dissipate heat. This will result in increases in their energy expenditure and water loss. As *E*. *wahlbergi* also slept relatively little in the laboratory, it is unclear how the loss of sleep due to hot days affects them and if they suffer from sleep deprivation. Lack of data on sleep behaviour by bats in general suggests that more work is required for comparison with results of those in other regions and species to improve our understanding of the ecophysiology, particularly given predicted climate change scenarios.
